# A non-ionotropic activity of NMDA receptors contributes to glycine-induced neuroprotection in cerebral ischemia-reperfusion injury

**DOI:** 10.1038/s41598-017-03909-0

**Published:** 2017-06-15

**Authors:** Juan Chen, Rong Hu, Huabao Liao, Ya Zhang, Ruixue Lei, Zhifeng Zhang, Yang Zhuang, Yu Wan, Ping Jin, Hua Feng, Qi Wan

**Affiliations:** 1 Department of Physiology, Collaborative Innovation Center for Brain Science, School of Basic Medical Sciences, School of Medicine, Wuhan University, 185 Donghu Street, Wuhan, Hubei 430071 China; 2 Department of Neurology, the Central Hospital of Wuhan, Tongji Medical College of Huazhong University of Science & Technology, 26 Shengli Street, Wuhan, 430014 China; 3 Department of Neurosurgery, Southwest Hospital, Chongqing, 400038 China; 4 Institute of Neuroregeneration & Neurorehabilitation, Qingdao University School of Medicine, 308 Ningxia Street, Qingdao, 266071 China

## Abstract

NMDA receptor (NMDAR) is known for its ionotropic function. But recent evidence suggests that NMDAR also has a non-ionotropic property. To determine the role of non-ionotropic activity of NMDARs in clinical relevant conditions, we tested the effect of glycine, a co-agonist of NMDARs, in rat middle cerebral artery occlusion (MCAO), an animal model of cerebral ischemia-reperfusion injury after the animals were injected with the NMDAR channel blocker MK-801 and the glycine receptor antagonist strychnine. We show that glycine reduces the infarct volume in the brain of ischemic stroke animals pre-injected with MK-801 and strychnine. The effect of glycine is sensitive to the antagonist of glycine-GluN1 binding site and blocked by Akt inhibition. In the neurobehavioral tests, glycine improves the functional recovery of stroke animals pre-injected with MK-801 and strychnine. This study suggests that glycine-induced neuroprotection is mediated in part by the non-ionotropic activity of NMDARs via Akt activation in cerebral ischemia-reperfusion injury.

## Introduction

The ionotropic glutamate receptors mediate the vast majority of excitatory neurotransmission in the mammalian central nervous system (CNS)^1, 2^. The N-methyl-D-aspartate receptor (NMDAR) is a subtype of ionotropic glutamate receptors that are ligand-gated Ca^2+^-permeable channels. NMDARs consist of GluN1, GluN2 (GluN2A-GluN2D) and GluN3 (GluN3A-GluN3B) subunits. In the CNS, GluN2A- and GluN2B-containing NMDARs (GluN2ARs and GluN2BRs) are the major combinations of NMDARs^2, 3^. Activation of GluN2ARs and GluN2BRs requires the binding of agonist glutamate to GluN2 subunits and the co-agonist glycine to GluN1 subunits, which play essential roles in synaptic plasticity, neural development and glutamate-induced neurotoxicity^4, 5^.

The GluN2 subunits confer distinct roles of NMDAR subtypes and link them with different intracellular signaling pathways^6, 7^. It has been reported that GluN2BR-mediated neurotoxicity induces neuronal death, and that enhancement of GluN2AR activity promotes neuronal survival^8, 9^. However, the molecular mechanisms underlying the differential effects of GluN2ARs and GluN2BRs in neuronal survival and death are not fully understood^10^.

Recent studies demonstrate that NMDARs have non-ionotropic activity that confers both physiological and pathophysiological effects^11–16^. For example, ligand binding to NMDARs is sufficient to induce long-term depression (LTD), but does not require ion flow through NMDARs^12^. A non-ionotropic activity is found to be mediated through GluN2BR and is required for β-amyloid–induced synaptic depression^13^. The non-ionotropic activity of NMDARs is shown to drive structural shrinkage at spiny synapses and couple Src family kinases to pannexin-1 in excitotoxic injury^14^. We recently report that glycine potentiates AMPA receptor function through non-ionotropic activation of GluN2ARs^15^. We also show that glycine protects against glutamate neurotoxicity-induced neuronal injury in cultured cortical neurons independent of the channel activity of GluN2ARs^16^. As glutamate-induced neurotoxicity plays critical role in many neurological disorders including ischemic stroke, in the present study we tested the effect of glycine in rat middle cerebral artery occlusion (MCAO)^17, 18^, an animal model of cerebral ischemia-reperfusion injury after the animals were injected with the NMDAR channel blocker MK-801 and the glycine receptor antagonist strychnine. We demonstrate that glycine treatment reduces the infarct volume and improves the functional recovery of stroke animals, suggesting that glycine-induced neuroprotection is mediated in part by the non-ionotropic activity of NMDARs in cerebral ischemia-reperfusion injury.

## Results

### Glycine protects against ischemia-reperfusion injury through non-ionotropic activity of NMDARs

**Figure 1:**
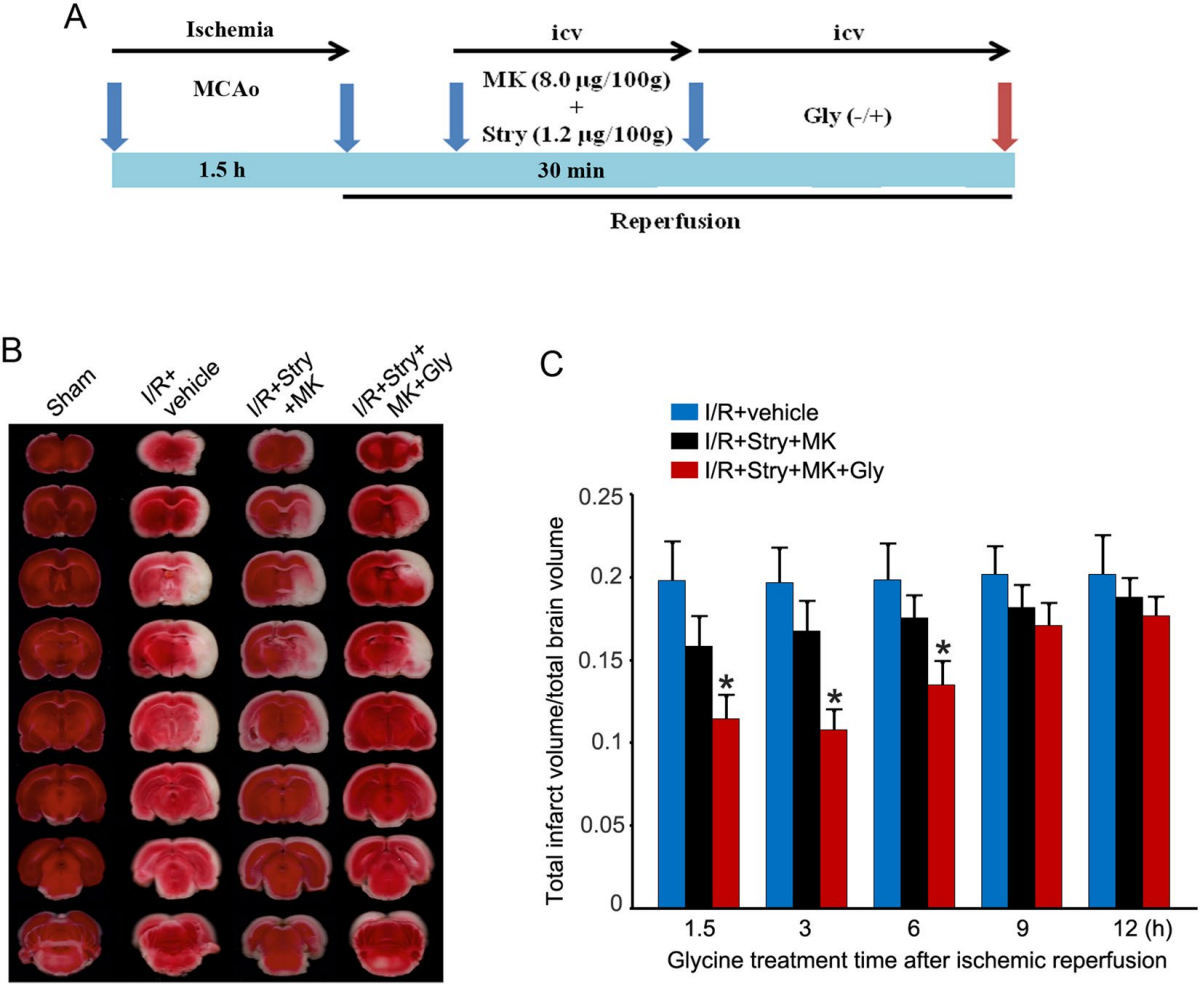
Glycine treatment reduces the infarct volume of ischemic brain independent of glycine receptor activation and the channel activity of NMDARs. (**A**) A schematic diagram showing rat cerebral ischemia-reperfusion injury and glycine treatment procedure. (**B**) Sample images of TTC strained-brain sections collected at 24 h after ischemia onset. Glycine (100 µg/100 g, icv) was administered at 3 h following ischemia-reperfusion (I/R). At 30 min prior to glycine injection, MK-801 (8.0 µg/100 g, icv) and strychnine (1.2 µg/100 g, icv) were injected. (**C**) Summarized quantification data indicate that glycine treatment at 1.5, 3, or 6 h following I/R reduces infarct volume after glycine receptors and NMDARs are inhibited (n = 10 for each group; ANOVA test, *P < 0.05 vs. I/R + Stry + MK). Stry: Strychnine; Gly: glycine; MK: MK-801.

We recently reported that glycine protects against glutamate neurotoxicity-induced neuronal injury through non-ionotropic activity of GluN2ARs^16^. To determine the functional effect of non-ionotropic activity of NMDARs, we tested whether the observed glycine effect *in vitro* occurred in rat MCAO model *in vivo*. Glycine (100 µg/100 g) was administered into the lateral ventricles at 1.5, 3, 6, 9 or 12 h following ischemia-reperfusion (Fig. 1 and S1). To suppress both the channel activity of NMDARs and the activation of glycine receptors, at 30 min before glycine injection we injected MK-801 (8.0 µg/100 g)^19, 20^ and strychnine (1.2 µg/100 g) into lateral ventricles^21–24^, which was referred to as NMDAR channel inactivation procedure (Fig. 1A). TTC staining assay revealed that treatment of glycine at 1.5, 3 or 6 h after ischemia-reperfusion significantly decreased the infarct volume at 24 h after ischemia onset compared with the groups with the injection of MK-801+ strychnine at the same time points (Fig. 1B and C). In the same experimental conditions, we also demonstrated that glycine (100 µg/100 g) reduced the infarct volume in a mouse model of MCAO at 24 h after ischemia onset (Fig. S2A and S2B).

**Figure 2:**
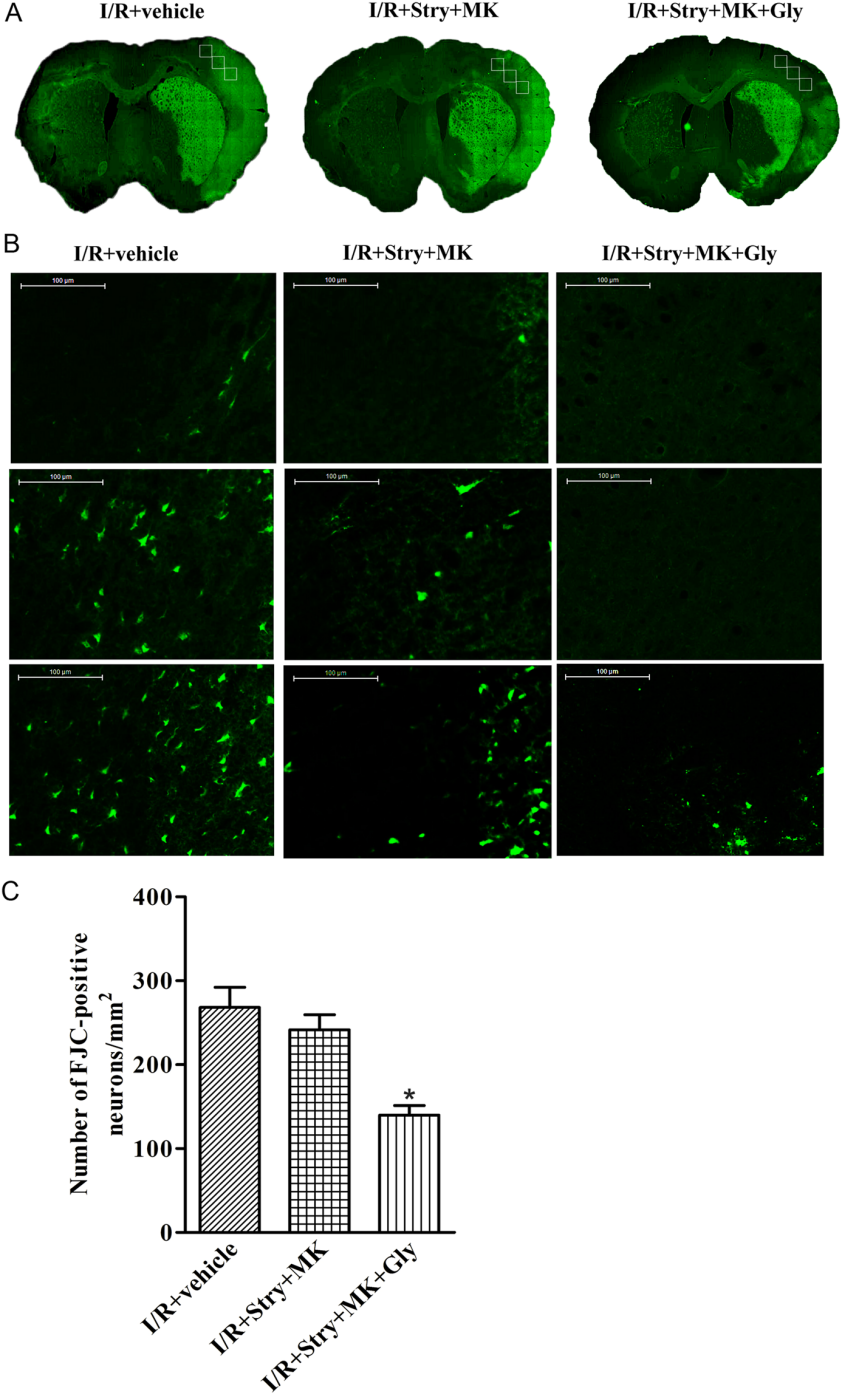
Glycine treatment reduces cell loss in the ischemic brain where NMDAR channel activity and glycine receptors are inhibited. (**A**) Sample images of FJC strained-brain sections collected at 3 d after ischemia onset. Glycine (100 µg/100 g, icv) was administered at 3 h following I/R. At 30 min prior to glycine injection, MK-801 (8.0 µg/100 g, icv) and strychnine (1.2 µg/100 g, icv) were injected. (**B**) The bottom panel is a higher magnification of three regions in the upper panel marked with a frame. Bar = 100 μm. (**C**) Quantitative analysis of FJC-positive degenerating neurons in the ischemic brain area at 3 d after ischemia onset (n = 6, ANOVA test, *P < 0.05 vs. I/R + Stry + MK). Stry: Strychnine; Gly: glycine; MK: MK-801.

To determine the neuroprotective effect of glycine in a late stage after ischemia-reperfusion, we performed Fluoro-Jade C (FJC) straining at 3 days after rat MCAO onset. Glycine (100 µg/100 g, icv) was administered at 3 h following ischemia-reperfusion. At 30 min prior to glycine injection, MK-801 (8.0 µg/100 g, icv) and strychnine (1.2 µg/100 g, icv) were injected. We showed that the number of FJC-positive degenerating neurons in the ischemic area in glycine treatment group was remarkably lower than the group without glycine injection (Fig. 2A–C). Together, these results suggest that glycine is neuroprotective in ischemia-reperfusion injury and the neuroprotection is mediated by the non-ionotropic activity of NMDARs.

### Glycine-GluN1 binding mediates glycine-induced neuroprotection after glycine receptors and the channel activity of NMDARs are suppressed

**Figure 3:**
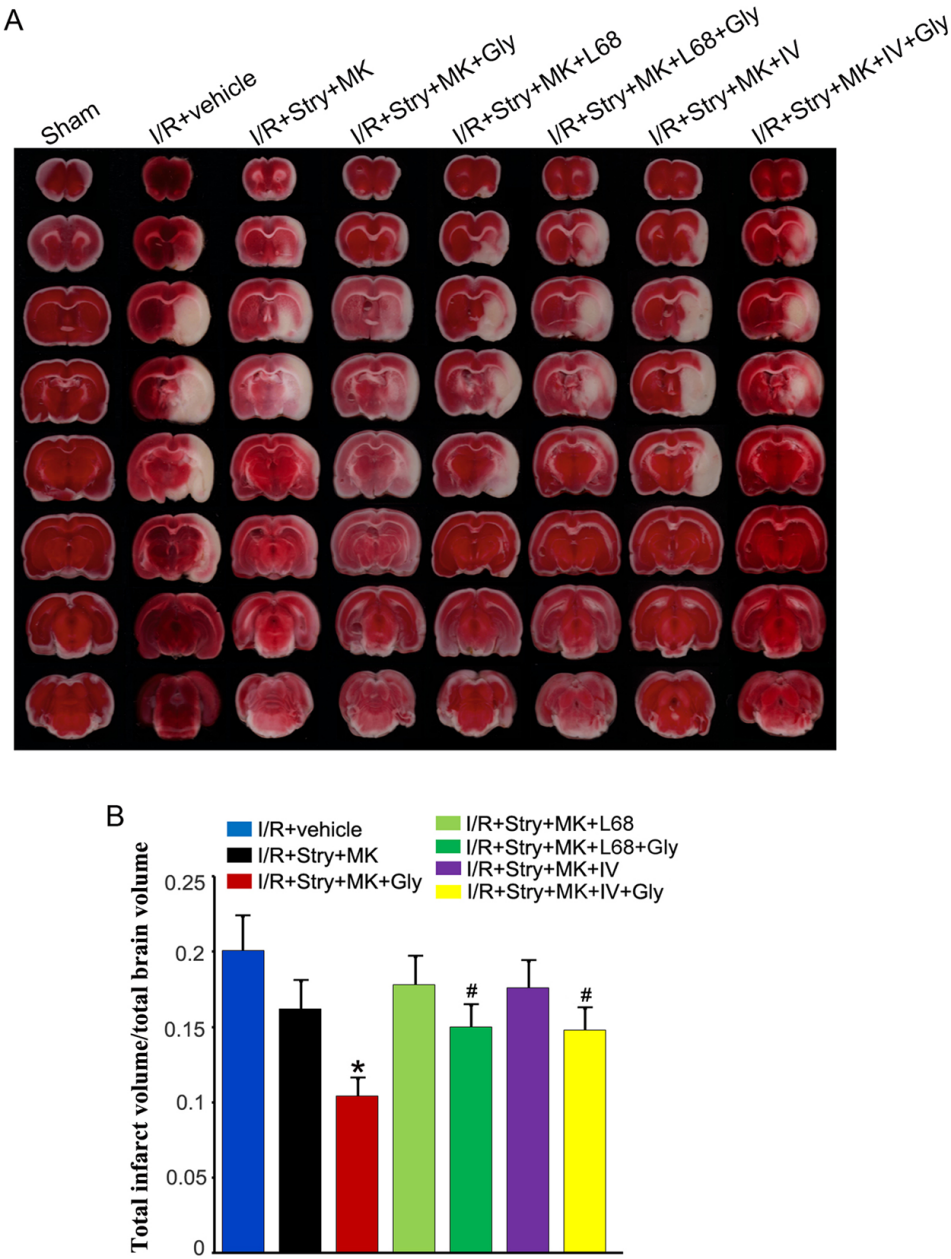
Glycine-GluN1 binding antagonist and Akt inhibitor attenuate the effect of glycine in reducing the infarct volume after glycine receptors and the channel activity of NMDARs are inhibited. (**A**) Sample images of TTC strained-brain sections collected at 24 h after ischemia onset. Glycine (100 µg/100 g, icv) was administered at 3 h following I/R. Glycine-GluN1 binding antagonist L-689560 (L68; 0.1 mg/100 g, ip) or Akt inhibitor IV (100 µM, 2 µl, icv) is injected with MK-801 and strychnine at 30 min before glycine injection. (**B**) Summarized quantification data indicate that glycine-GluN1 binding antagonist and Akt inhibitor treatment attenuate the effect of glycine in reducing the infarct volume after glycine receptors and NMDARs are inhibited (n = 6, ANOVA test, *P < 0.05 vs. I/R + Stry + MK; ^#^ *P* < 0.05 vs. I/R + Stry + MK + Gly). Stry: Strychnine; Gly: glycine; MK: MK-801.

To provide further evidence for how glycine induced neuroprotection following ischemia-reperfusion injury, we examined the effect of glycine-GluN1 binding site antagonist L-689560 in the rat MCAO model. L-689560 (0.1 mg/100 g, ip) was injected with MK-801 and strychnine at 30 min prior to glycine treatment as described previously^25–27^. Glycine (100 µg/100 g) was administered into the lateral ventricles at 3 h after ischemia-reperfusion. We showed that L-689560 significantly reduced the neuroprotective effect of glycine after glycine receptors and the channel activity of NMDARs are suppressed (Fig. 3A and B). These data indicate that glycine acts on glycine-GluN1 binding site to elicit non-ionotropic activation of NMDARs and exert neuroprotective effect in ischemic stroke animals.

### Glycine-elicited non-ionotropic activity of NMDARs confers neuroprotection through Akt activation after ischemia-reperfusion injury

**Figure 4:**
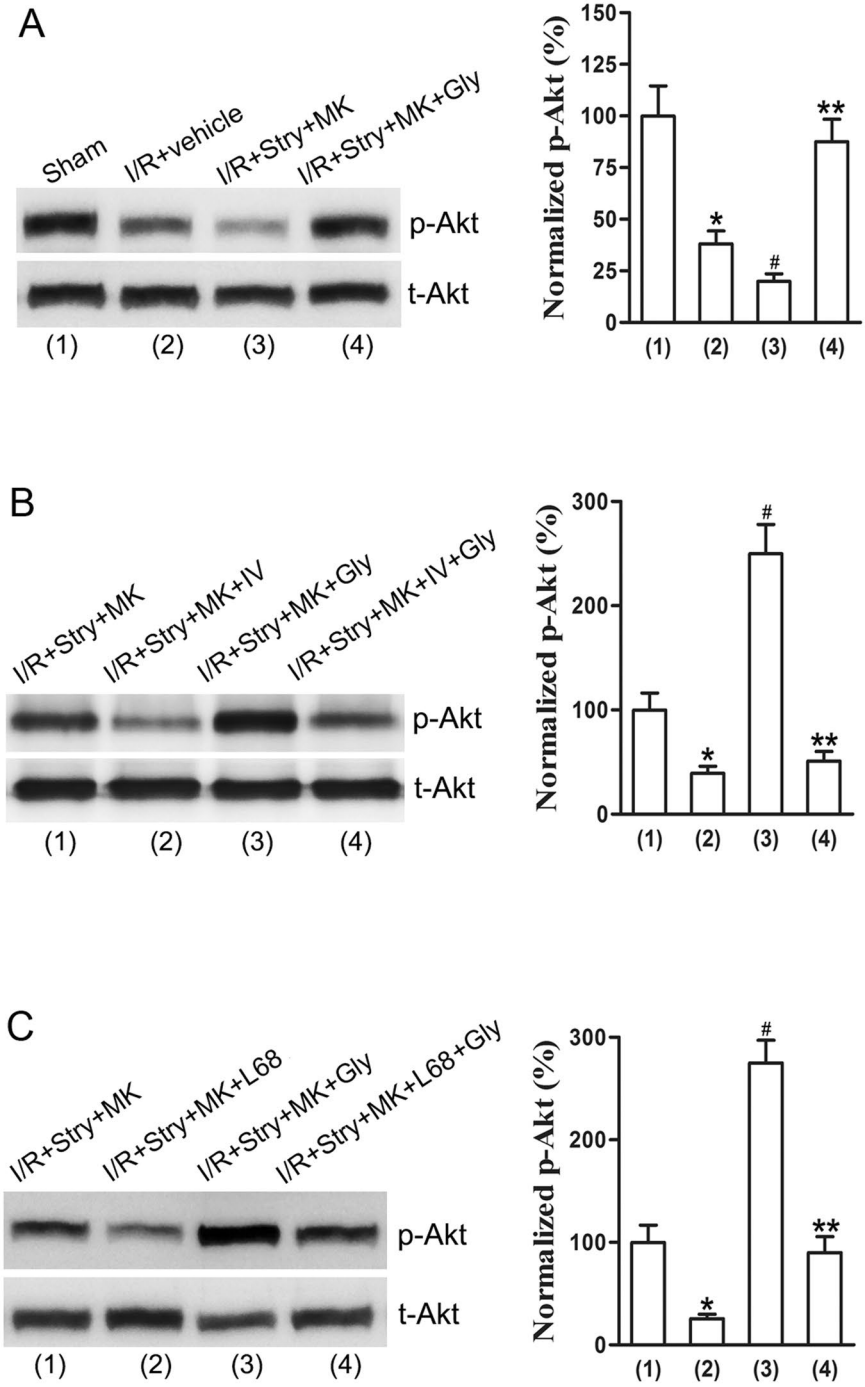
Glycine treatment prevents p-Akt reduction in ischemic penumbra where NMDAR channel activity and glycine receptors are inhibited. (**A**) Glycine (100 µg/100 g, icv) treatment at 3 h following I/R prevents p-Akt reduction in ischemic penumbra where NMDAR channel activity and glycine receptors are inhibited (n = 6 animals for each group; ANOVA test, *P < 0.05 vs. Sham; ^#^ *P* < 0.05 vs. I/R + vehicle; **P < 0.05 vs. I/R + Stry + MK). Samples were collected at 24 h after ischemia onset. (**B**) Akt inhibitor IV (100 µM, 2 µl, icv) prevents glycine (100 µg/100 g, icv)-induced increase of p-Akt in ischemic penumbra where NMDAR channel activity and glycine receptors are inhibited (n = 6, ANOVA test, *P < 0.05 vs. I/R + Stry + MK; ^#^ *P* < 0.05 vs. I/R + Stry + MK; **P < 0.05 vs. I/R + Stry + MK + Gly). The IV was injected with MK-801 and strychnine at 30 min before glycine injection. Samples were collected at 24 h after ischemia onset. (**C**) The L-689560 (0.1 mg/100 g, ip) prevents glycine (100 µg/100 g, icv)-induced increase of p-Akt in ischemic penumbra where NMDAR channel activity and glycine receptors are inhibited (n = 6, ANOVA test, *P < 0.05 vs. I/R + Stry + MK; ^#^ *P* < 0.05 vs. I/R + Stry + MK; **P < 0.05 vs. I/R + Stry + MK + Gly). The L-689560 was injected with MK-801 and strychnine at 30 min before glycine injection. Samples were collected at 24 h after I/R. Stry: Strychnine; Gly: glycine; MK: MK-801; L-68: L-689560.

Our data thus far support the notion that glycine elicits a non-ionotropic activity of NMDARs to confer neuroprotection after ischemia-reperfusion injury. To uncover downstream signaling that mediates the effect of non-ionotropic activity of NMDARs in ischemia-reperfusion injury, we tested the role of Akt in the rat MCAO model. Akt inhibitor IV (100 μM, 2 μl, icv) was injected with MK-801 and strychnine at 30 min prior to glycine treatment as described above^28^. Glycine (100 µg/100 g) was administered into the lateral ventricles at 3 h after ischemia-reperfusion. As shown in Fig. 3A and B, Akt inhibitor IV reduced the neuroprotective effect of glycine after glycine receptors and the channel activity of NMDARs are suppressed. In the same experimental conditions, we showed that L-689560 and IV blocked glycine-induced increase of Akt phosphorylation in the MCAO model (Fig. 4A–C). Thus, Akt is a downstream signal to mediate the neuroprotective role of non-ionotropic activation of NMDARs by glycine in ischemic stroke animals.

### Glycine promotes functional recovery of ischemic stroke animals through non-ionotropic activity of NMDARs

**Figure 5:**
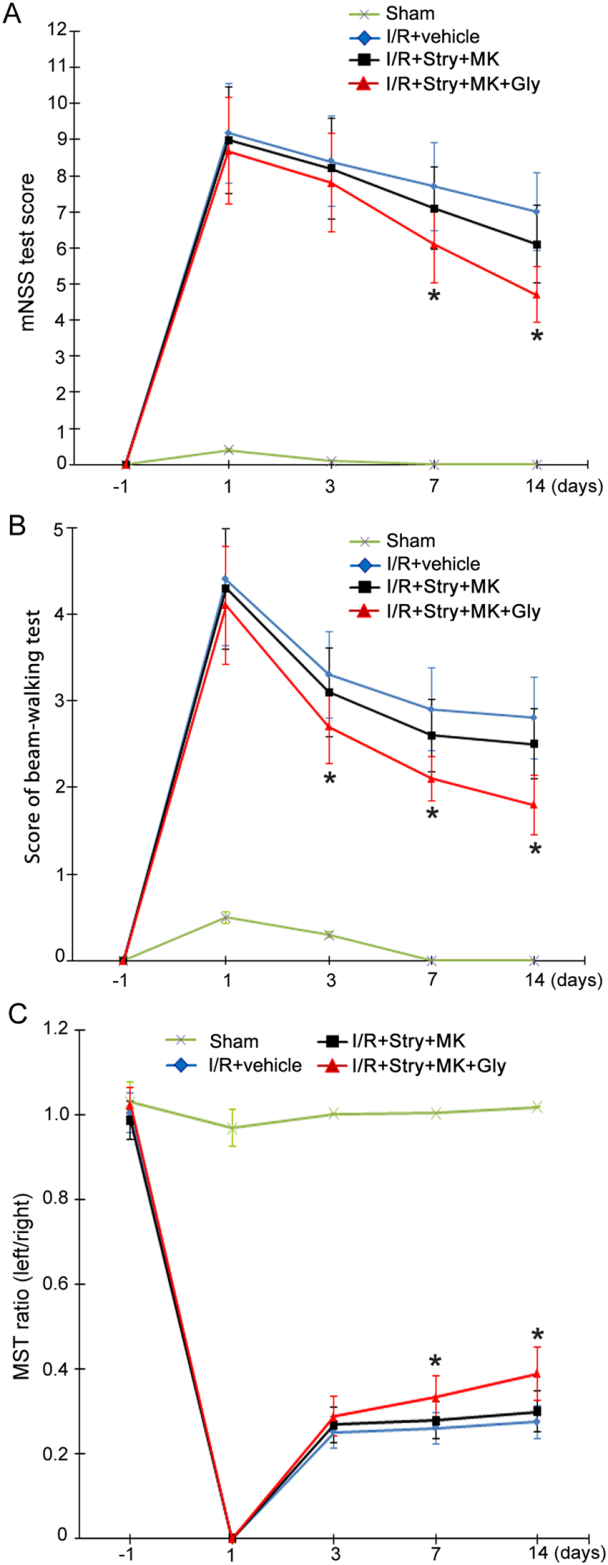
Glycine promotes functional recovery of ischemic stroke animals independent of glycine receptor activation and the channel activity of NMDARs. For all the three experiments, Glycine (100 µg/100 g, icv) was administered at 3 h following I/R. At 30 min prior to glycine injection, MK-801 (8.0 µg/100 g, icv) and strychnine (1.2 µg/100 g, icv) were injected. (**A**) Animals treated with glycine have lower scores of mNSS test at day 7 and 14 after I/R compared with I/R + Stry + MK group (n = 10; ANOVA test, *P < 0.05 vs. I/R + Stry + MK). (**B**) Animals treated with glycine has lower scores of beam-walking test at day 3, 7 and 14 after I/R compared with I/R + Stry + MK group (n = 10; ANOVA test, *P < 0.05 vs. I/R + Stry + MK). (**C**) Animals treated with glycine has higher ratio in MST test at day 7 and 14 after I/R compared with I/R + Stry + MK group (n = 10; ANOVA test, *P < 0.05 vs. I/R + Stry + MK). Stry: Strychnine; MK: MK-801; Gly: glycine.

A battery of neurobehavioral tests including modified neurological severity scores (mNSS) test, beam-walking test and modified sticky-tape (MST) test were performed to further test the functional role of glycine-induced neuroprotection (Tables 1 and 2)^29, 30^. The neurological function of stroke animals was evaluated one day before MCAO, and 1, 3, 7 and 14 days after MCAO. Glycine (100 µg/100 g, icv) was injected at 3 h after ischemia-reperfusion. At 30 min prior to glycine injection we injected MK-801 (8.0 µg/100 g) and strychnine (1.2 µg/100 g) into the lateral ventricles. These tests were performed by the investigator who was blinded to the experimental groups. Our data showed that compared with rats treated with strychnine and MK-801 treatment (I/R + Stry + MK group), rats treated with glycine (I/R + Stry + MK + Gly group) had significantly lower scores of mNSS test at day 7 and 14 after MCAO (Fig. 5A), lower scores of beam-walking test at day 3, 7 and 14 after MCAO (Fig. 5B), and higher ratio of MST test at day 7 and 14 after MCAO (Fig. 5C). These results provide functional evidence for the role of non-ionotropic activity of NMDARs in mediating the neuroprotective effect of glycine.

**Table 1:** Modified Neurological Severity Score (mNSS).

	Points
Motor tests	
Raising rat by tail	3
Flexion of forelimb	1
Flexion of hindlimb	1
Head moved > 10° to vertical axis within 30 s	1
Placing rat on floor (normal = 0; maximum = 3)	3
Normal walk	0
Inability to walk straight	1
Circling toward the paretic side	2
Fall down to the paretic side	3
Sensory tests	2
Placing test (visual and tactile test)	1
Proprioceptive test (deep sensation, pushing paw against table edge to stimulate limb muscles)	1
Beam balance tests (normal = 0; maximum = 6)	6
Balances with steady posture	0
Grasps side of beam	1
Hugs beam and 1 limb falls down from beam	2
Hugs beam and 2 limbs fall down from beam, or spins on beam (å 60 s)	3
Attempts to balance on beam but falls off (å 40 s)	4
Attempts to balance on beam but falls off (å 20 s)	5
Falls off; No attempt to balance or hang on to beam (20 s)	6
Reflex absence and abnormal movements	4
Pinna reflex (head shake when auditory meatus is touched)	1
Corneal reflex (eye blink when cornea is lightly touched with cotton)	1
Startle reflex (motor response to a brief noise from snapping a clipboard paper)	1
Seizures, myoclonus, myodystony	1
Maximum points	18

One point is awarded for the inability to perform the tasks or for lack of a tested reflex, 13–18, severe injury; 7–12, moderate injury; 1–6, mild injury.

**Table 2:** The Beam Walk Test Scoring Criteria.

Complex Neuromotor Function	Score	Time on Beam
	0	−4 s or less.
	1.0	−5 to 7 s.
	2.0	−8 to 10 s.
	3.0	−11 to 15 s.
	4.0	-greater than 15 s.
	5.0	-not able to run
Maximum Score	**5**

### Glycine-GluN1 binding mediates glycine-induced functional recovery of ischemic stroke animals after glycine receptors and the channel activity of NMDARs are suppressed

**Figure 6:**
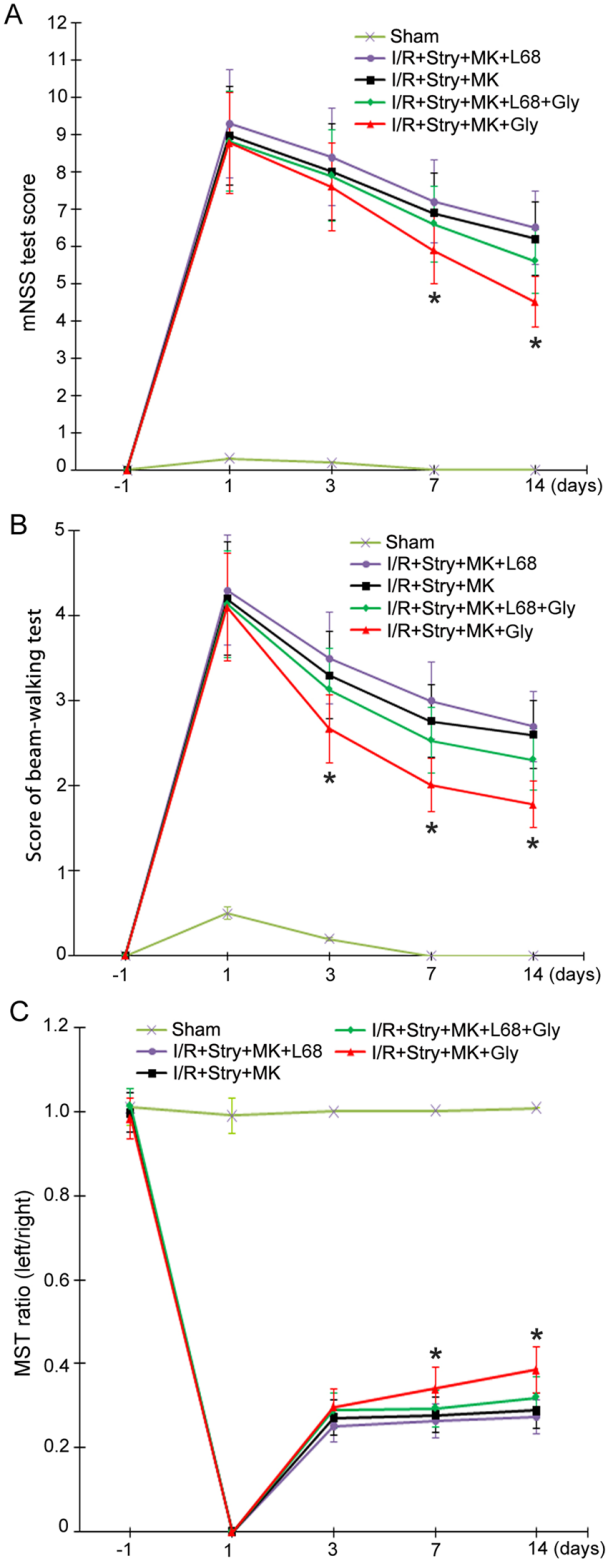
Glycine-GluN1 binding site antagonist prevents glycine-induced functional recovery of ischemic stroke animals. For all the three experiments, Glycine (100 µg/100 g, icv) was administered at 3 h following I/R. Glycine-GluN1 binding antagonist L-689560 (L68; 0.1 mg/100 g, ip) is injected with MK-801 and strychnine at 30 min before glycine injection. (**A**) Animals treated with glycine (100 µg/100 g, icv) has lower scores of mNSS test at day 7 and 14 compared with I/R + Stry + MK + L68 + Gly group (n = 10; ANOVA test, *P < 0.05, vs. I/R + Stry + MK + L68 + Gly). (**B**) Animals treated with glycine has lower scores of beam-walking test at day 3, 7 and 14 compared with I/R + Stry + MK + L68 + Gly group (n = 10; ANOVA test, *P < 0.05, vs. I/R + Stry + MK + L68 + Gly). (**C**) Animals treated with glycine (100 µg/100 g, icv) treatment has higher ratio in MST test at day 7 and 14 compared with I/R + Stry + MK + L68 + Gly group (n = 10; ANOVA test, *P < 0.05, vs. I/R + Stry + MK + L68 + Gly). Stry: Strychnine; Gly: glycine; MK: MK-801.

To determine whether glycine-GluN1 binding mediates glycine-induced functional recovery in the ischemic stroke animals, in the same experimental conditions, L-689560 (0.1 mg/100 g, ip) was injected with MK-801 (8.0 µg/100 g) and strychnine (1.2 µg/100 g) at 30 min prior to glycine treatment. As shown in Fig. 6, L-689560 prevented glycine-induced recovery in stroke animals after glycine receptors and NMDAR channel activities were inhibited, suggesting that glycine binds to GluN1 site to elicit non-ionotropic activity of NMDARs and promote functional recovery in ischemic stroke animals.

### Glycine-elicited non-ionotropic activity of NMDARs confers functional recovery in ischemic stroke animals through Akt activation

**Figure. 7:**
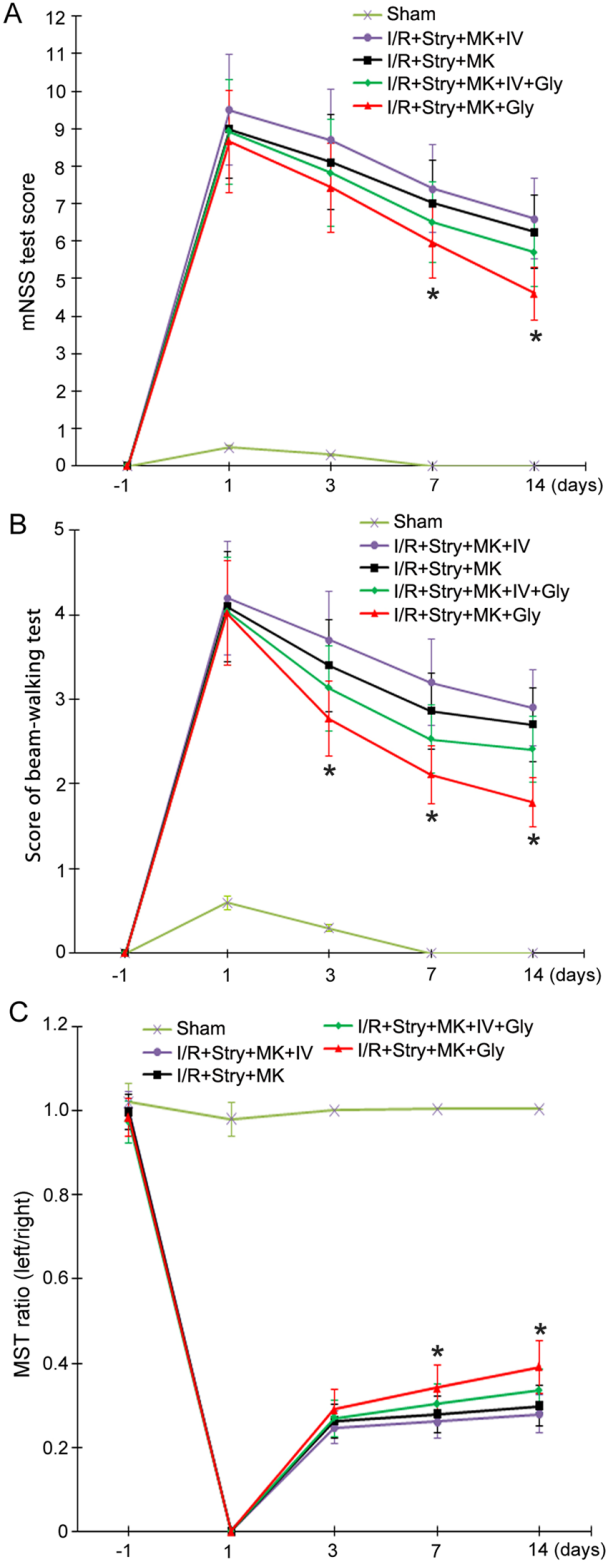
Akt inhibitor IV prevents glycine-induced functional recovery of ischemic stroke animals. For all the three experiments, Glycine (100 µg/100 g, icv) was administered at 3 h following I/R. Akt inhibitor IV (100 µM, 2 µl, icv) is injected with MK-801 and strychnine at 30 min before glycine injection. (**A**) Animals treated with glycine (100 µg/100 g. icv) has lower scores of mNSS test at day 7 and 14 compared with I/R + Stry + MK + IV + Gly group (n = 10; ANOVA test, *P < 0.05, vs. I/R + Stry + MK + IV + Gly). (**B**) Animals treated with glycine has lower scores of beam-walking test at day 3, 7 and 14 compared with I/R + Stry + MK + IV + Gly group (n = 10; ANOVA test, *P < 0.05, vs. I/R + Stry + MK + IV + Gly). (**C**) Animals treated with glycine (100 µg/100 g, icv) treatment has higher ratio in MST test at day 7 and 14 compared with I/R + Stry + MK + IV + Gly group (n = 10; ANOVA test, *P < 0.05, vs. I/R + Stry + MK + IV + Gly). Stry: Strychnine; Gly: glycine; MK: MK-801.

To provide evidence for the role of Akt activation in mediating glycine-induced functional recovery in the ischemic stroke animals, we tested the effect of Akt inhibition in the same experimental conditions as described above^31, 32^. Akt inhibitor IV (100 μM, 2 μl, icv) was injected with MK-801 (8.0 µg/100 g) and strychnine (1.2 µg/100 g) at 30 min prior to glycine treatment. We found that Akt inhibitor IV blocked glycine-induced recovery of stroke animals after glycine receptors and NMDAR channel activities were inhibited (Fig. 7). These results support the conclusion that glycine triggers a non-ionotropic activation of NMDARs and induces a functional recovery in ischemic stroke animals via Akt activation.

## Discussion

Glycine is a co-agonist of ionotropic NMDARs^1^. We have previously shown that glycine enhances the activation of cell survival-promoting kinase Akt in cultured cortical neurons in which both the channel activity of NMDARs and the glycine receptors are pre-inhibited^16^. The effect of glycine is reduced by shRNA-mediated knockdown of GluN2ARs, suggesting that a non-ionotropic activity of GluN2ARs mediates glycine-induced Akt activation. In support of this finding, glycine enhances Akt activation in HEK293 cells over-expressing GluN2ARs. The effect of glycine on Akt activation is sensitive to the antagonist of glycine-GluN1 binding site. As a functional consequence, glycine protects against glutamate excitotoxicity-induced neuronal death through the non-ionotropic activity of GluN2ARs and the neuroprotective effect is attenuated by Akt inhibition. Thus, we demonstrated a role of glycine in eliciting a non-ionotropic activity of GluN2ARs to confer neuroprotection via Akt activation *in vitro* ^16^.

Since glutamate-induced neurotoxicity is known to mediate neuronal death in ischemia-reperfusion injury, according to our *in vitro* findings we tested the effect of glycine in the rat middle cerebral artery occlusion (MCAO), an animal model of cerebral ischemia-reperfusion injury after the animals were injected with the NMDAR channel blocker MK-801 and the glycine receptor antagonist strychnine^1, 8, 33^. We demonstrate that glycine treatment reduces the infarct volume and improves the functional recovery of stroke animals. These data support our *in vitro* finding that glycine-induced neuroprotection is mediated in part by the non-ionotropic activity of NMDARs and provide a functional evidence for the neuroprotective role of non-ionotropic activity of NMDARs in cerebral ischemia-reperfusion injury. Due to the technical limitation in the *in vivo* stroke model, we were unable to provide direct evidence to determine whether the non-ionotropic activity of NMDARs is mediated by GluN2ARs and whether the non-ionotropic activity of GluN2ARs mediates glycine-induced neuroprotection in the animal model of cerebral ischemia-reperfusion injury. However, we showed that both glycine-GluN1 binding site antagonist and Akt inhibitor attenuated glycine-induced neuroprotection in the ischemic brain tissues where NMDAR channel activities and glycine receptors were inhibited, which is consistent with our *in vitro* findings. These data provide indirect evidence to support the role of non-ionotropic activity of GluN2ARs mediates glycine-induced neuroprotection in cerebral ischemia-reperfusion injury.

In the experimental conditions in which NMDAR channel activity and glycine receptors were inhibited, we found that glycine treatment prevented neuronal loss in ischemic penumbra at 7 d after ischemia-reperfusion, and that glycine promoted functional recovery of stroke animals. These results provide further evidence to support glycine as a potential neuroprotectant in stroke therapy.

How the non-ionotropic activity of NMDARs enhances Akt activation is unclear^34, 35^. It is possible that the C-terminal domain of NMDARs may mediate the effect of glycine on Akt activation. Indeed, recent study provides evidence that agonist binding to the NMDAR drives movement of its cytoplasmic domain independent of ion flow, and changes the interaction between the cytoplasmic domain and its downstream signaling such as protein phosphatase 1 (PP1) and calcium-calmodulin-dependent protein kinase II (CaMKII), which regulates synaptic depression^36, 37^.

Akt deactivation is believed to be a causal mediator of cell death^38^. Enhancement of Akt activity exerts pro-survival effect in neuronal injury and neurodegenerative diseases^31, 39^. In this study, we identify Akt as a downstream neuroprotective signal of glycine that activates non-ionotropic NMDARs. Akt is known to influence neuronal survival through activation or inhibition of substrates. For example, activated Akt promotes survival through phosphorylation of transcription factors forkhead/FOXO, NF-κ B and mdm2 or through phosphorylation of Bcl-2 family members Bad and Bim^23^. Further study is needed to determine which Akt-dependent signal pathway mediates the activation of non-ionotropic NMDARs by glycine.

NMDAR-mediated neurotoxicity induces neuronal death and neurodegeneration in various CNS disorders including ischemic stroke, traumatic brain injury and neurodegenerative diseases^4, 9, 40–42^. However, the use of NMDAR antagonists as neuroprotective agents was disappointing in clinical trials^10^. A simple possibility is that these antagonists, while suppressing NMDAR-mediated neurotoxicity, block the biological and/or neural survival-promoting effects of NMDARs. Thus, identification of molecular mechanisms by which specific NMDAR subtype selectively exerts its effect on neuronal survival or death would provide a critical basis for the development of potent therapy for CNS injuries and neurodegenerative diseases. Substantial evidence suggests that GluN2ARs and GluN2BRs play different role in neuronal survival or death^4, 5, 9^. But the underlying molecular mechanism remains unclear.

Recent studies demonstrate that NMDAR has non-ionotropic activity that plays an excitotoxic role^12, 13, 15–18^. A non-ionotropic activity is found to be mediated through GluN2BR and is required for β-amyloid–induced synaptic depression and synaptic loss^43^. The non-ionotropic activity of NMDARs is shown to drive structural shrinkage at spiny synapses and couple Src family kinases to pannexin-1 in excitotoxic injury^14^. These data provide new evidence for the involvement of GluN2BRs in mediating neurotoxicity. More recently, we report that glycine protects against glutamate neurotoxicity-induced neuron injury in cultured cortical neurons independent of the channel activity of GluN2ARs, indicating a non-ionotropic activation of GluN2ARs selectively by glycine^16^. The non-ionotropic activity of GluN2AR and GluN2BR explains in part why GluN2AR plays a different role than GluN2BR in neuronal survival. The neuroprotective effect of glycine in stroke animals observed in the present experimental conditions supports the involvement of non-ionotropic activity of GluN2ARs.

### Experimental Procedures

#### General methods

Adult male Sprague-Dawley (SD) rats were housed in cages on a 12 h light/dark cycle in a temperature-controlled room (23 °C–25 °C) with free access to food and water. Animals were allowed at least 3 days to acclimatize before experimentation. All animal use and experimental protocols were approved and carried out in compliance with the IACUC guidelines and the Animal Care and Ethics Committee of Wuhan University School of Medicine. Randomization was used to assign samples to the experimental groups, and to collect and process data. The experiments were performed by investigators blinded to the groups for which each animal was assigned.

#### Focal cerebral ischemia and infarct measurement

Transient focal cerebral ischemia was induced using the suture occlusion technique. Male Sprague-Dawley rats weighing 250–300 g were anesthetized with 4% isoflurane in 70% N_2_O and 30% O_2_ using a mask. The animals were anesthetized during surgery and drug injection at different time points after MCAO. The Control and Sham animals were all subjected to the same procedures. A midline incision was made in the neck, the right external carotid artery (ECA) was carefully exposed and dissected, and a 3–0 monofilament nylon suture was inserted from the ECA into the right internal carotid artery to occlude the origin of the right middle cerebral artery (MCA) (approximately 22 mm). After 90 minutes of occlusion, the suture was removed to allow reperfusion, the ECA was ligated, and the wound was closed. Sham-operated rats underwent identical surgery and/or intracerebroventricular injections except that the suture was inserted and withdrawn immediately. Rectal temperature was maintained at 37.0 ± 0.5 °C using a heating pad and heating lamp. Rats were killed at various times after reperfusion after being anesthetized, and the brains were removed for TTC (2,3,5 -triphenyltetrazolium chloride) staining. The brain was placed in a cooled matrix and 2 mm coronal sections were cut. Individual sections were placed in 10 cm petri dishes and incubated for 30 min in a solution of 2% TTC in phosphate buffered saline at 37 °C. The slices were fixed in 4% paraformaldehyde at 4 °C for 24 h. All image collection, processing and analysis were performed in a blind manner and under controlled environmental lighting. The scanned images were analyzed using image analysis software (Image-Pro Plus Version 6.0, USA). The infarct volume was calculated to correct for edema. The normal volume of contralateral hemisphere and the normal volume of ipsilateral hemisphere were measured, and the infarct percentage was calculated as % contralateral structure to avoid mismeasurement secondary to edema^44–46^.

#### Intracerebroventricular administration

For intracerebroventricular injections, the rats were placed on ear bars of a stereotaxic instrument under anesthesia. Drug infusion to the cerebral ventricle (from the bregma: posterior, 0.8 mm; lateral, 1.5 mm; depth, 3.5 mm) was performed using a 23-gauge needle attached via polyethylene tubing to a Hamilton microsyringe at a rate of 1.0 μl/min. The injection needle was left in place for an additional 5 min to allow diffusion, it was then slowly withdrawn^47, 48^.

#### Fluoro Jade–C (FJC) Staining

Mice were anesthetized with an overdose of isoflurane, followed by intracardiac perfusion with normal saline, followed by 4% paraformaldehyde post-fixed at 4 °C for 24 h, and then transferred into 30% sucrose solution in 0.1 mol/L phosphate buffer at 4 °C for 72 h. The brains were removed by decapitation and kept overnight in 4% paraformaldehyde solution at 4 °C. Brains were cut into 20 μm coronal sections by a Leica VT1000S vibratome (Leica Micro-systems AG, Nussloch, Germany). FJC labelling was performed using a standard protocol with modification^49^. Slides were immersed in 1% sodium hydroxide in 80% ethanol for 5 min, followed by rinsing in 70% ethanol for 2 min, in distilled water for 2 min, and then incubated in 0.06% potassium permanganate solution for 10 min. After 2 min in distilled water, the slides were transferred into 0.0001% solution of FJC (Sigma Aldrich, USA) which was dissolved in 0.1% acetic acid. Slides were rinsed with distilled water for 1 min, three times, and then dried at 50 °C for 5 min, immersed in xylene for 1 min. The slices were mounted with DPX media (Sigma Aldrich, USA). The sections were photographed by a blinded investigator using an Olympus fluorescent microscope (IX51, Olympus, Japan). Series of microphotographs were taken from three region of the ipsilateral cerebral cortex, with a ×20 objective and FJC-positive cells were counted by ImageJ software (ImageJ, USA). The data were expressed as cells/mm2.

#### Western blotting analysis

Rats were killed at the indicated time points, and right ischemia penumbra were isolated, beginning 3 mm from the anterior tip of the frontal lobe, a 4 mm thick coronal slice was chosen, then made a longitudinal cut (from top to bottom) approximately 1 mm from the midline through right hemisphere, and then made a transverse diagonal cut at approximately the “2 o’clock” position to separate the core from the penumbra. The tissues were homogenized in lysis buffer, which contained a protease and phosphatase inhibitor cocktail. Protein concentration was determined using a bicinchoninic acid protein assay (Thermo Scientific). Samples were separated by a sodium dodecyl sulfate–polyacrylamide gel and transferred to a polyvinylidene difluoride membrane (Millipore). For the detection of phospho-Akt, the samples prepared in the same day were used. The polyvinylidene difluoride membrane (Millipore, Bedford, MA, USA) was incubated with primary antibody against phospho-Akt (Ser473) (Cell Signaling Technology, Beverly, MA) or Akt (Cell Signaling). Primary antibodies were labeled with horseradish peroxidase-conjugated secondary antibody, and protein bands were imaged using SuperSignal West Femto Maximum Sensitivity Substrate (Pierce, Rockford, IL, USA). The EC3 Imaging System (UVP, LLC, Upland, CA) was used to obtained blot images directly from the polyvinylidene difluoride membrane. For the detection of total Akt, the same polyvinylidene difluoride membrane was stripped and then reprobed with primary antibody against total Akt (Cell Signaling Technology). The quantification of Western blot data was performed using ImageJ software^16, 50^.

#### Neurological Severity Scores

The rats were subjected to a modified neurological severity score (mNSS) test as reported previously (Table 1). These tests are a battery of motor, sensory, reflex, and balance tests, which are similar to the contralateral neglect tests in humans. Neurological function was graded on a scale of 0 to 18 (normal score, 0; maximal deficit score, 18)^51^.

#### Beam walk test

The beam walk test measures the animals’ complex neuromotor function. The animal was timed as it walked a (90 × 4 × 1.5 cm) beam. A box for the animal to feel safe was placed at one end of the beam. A loud noise was created to stimulate the animal to walk toward and into the box. Scoring was based upon the time it took the rat to go into the box. The higher the score, the more severe is the neurological deficit (Table 2)^52^.

#### Adhesive-removal test

A modified sticky-tape (MST) test was performed to evaluate forelimb function. A sleeve was created using a 3.0 × 1.0-cm piece of yellow paper tape and was subsequently wrapped around the forepaw so that the tape attached to itself and allowed the digits to protrude slightly from the sleeve. The typical response is for the rat to vigorously attempt to remove the sleeve by either pulling at the tape with its mouth or brushing the tape with its contralateral paw. The rat was placed in its cage and observed for 30 s. Two timers were started: the first ran without interruption and the second was turned on only while the animal attempted to remove the tape sleeve. The ratio of the left (affected)/right (unaffected) forelimb performance was recorded. The contralateral and ipsilateral limbs were tested separately. The test was repeated three times per test day, and the best two scores of the day were averaged. The lower the ratio, the more severe is the neurological deficit^29^.

#### Statistics

Student’s *t* test or ANOVA test was used where appropriate to examine the statistical significance of the differences between groups of data. Newman–Keuls tests were used for post-hoc comparisons when appropriate. All results are presented as mean ± SE. Significance was placed at *p* < 0.05.

## Electronic supplementary material

Supplementary information
