# Supplementary material for: A non-ionotropic activity of NMDA receptors contributes to glycine-induced neuroprotection in cerebral ischemia-reperfusion injury
Source: Sci Rep. 2017 Jun 15;7:3575. doi: 10.1038/s41598-017-03909-0 (PMC5472592; doi:10.1038/s41598-017-03909-0)
Supplement: Supplementary file 1 — Supplementary information [file 41598_2017_3909_MOESM1_ESM.pdf]

Supplementary information

A non-ionotropic activity of NMDA receptors contributes to glycine-induced neuroprotection in cerebral ischemia-reperfusion injury

Juan Chen[1,2 *], Rong Hu[3*], Huabao Liao[1], Ya Zhang[1] , Ruixue Lei[1], Zhifeng Zhang[1], Yang Zhuang[1], Yu Wan[1], Ping Jin[2], Hua Feng[3], Qi Wan[1,4]

[1]Department of Physiology, Collaborative Innovation Center for Brain Science, School of Basic Medical Sciences, School of Medicine, Wuhan University, 185 Donghu Street, Wuhan, Hubei, China 430071
[2]Department of Neurology, the Central Hospital of Wuhan, Tongji Medical College of Huazhong University of Science & Technology, 26 Shengli Street, Wuhan 430014, China
[3]Department of Neurosurgery, Southwest Hospital, Chongqing 400038, China
[4]Institute of Brain Research, Qingdao University School of Medicine, 308 Ningxia Street, Qingdao 266071, China

[*]These authors contributed equally to this work.

Correspondence should be addressed to: Dr. Qi Wan, Department of Physiology, School of Basic Medical Sciences, Wuhan University School of Medicine, 185 Donghu Street, Wuhan, China 430071. Email: qwan@whu.edu.cn; Juan Chen, Department of Neurology, the Central Hospital of Wuhan, Tongji Medical College of Huazhong University of Science & Technology, Wuhan 430014, China. Email: chenjuan0828@163.com

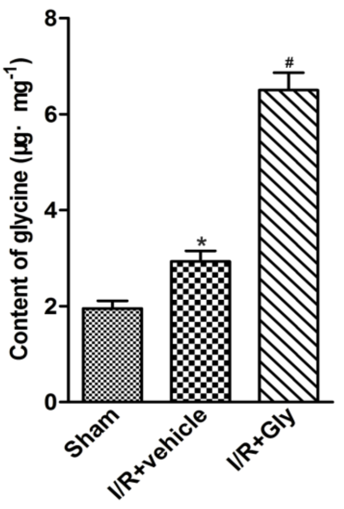

Figure S1. Intracerebroventricular injection of glycine leads to the elevation of glycine in the ischemic brain tissue. Intravenous injection of (100 μg/100 g) at 3.0 h after ischemia-reperfusion increases the levels of glycine in the ischemic brain tissue at 30 min following glycine injection (n=6, *P<0.05 vs. Sham; $^{\#}$P<0.05 vs. I/R+Vehicle; ANOVA test). Gly: glycine.

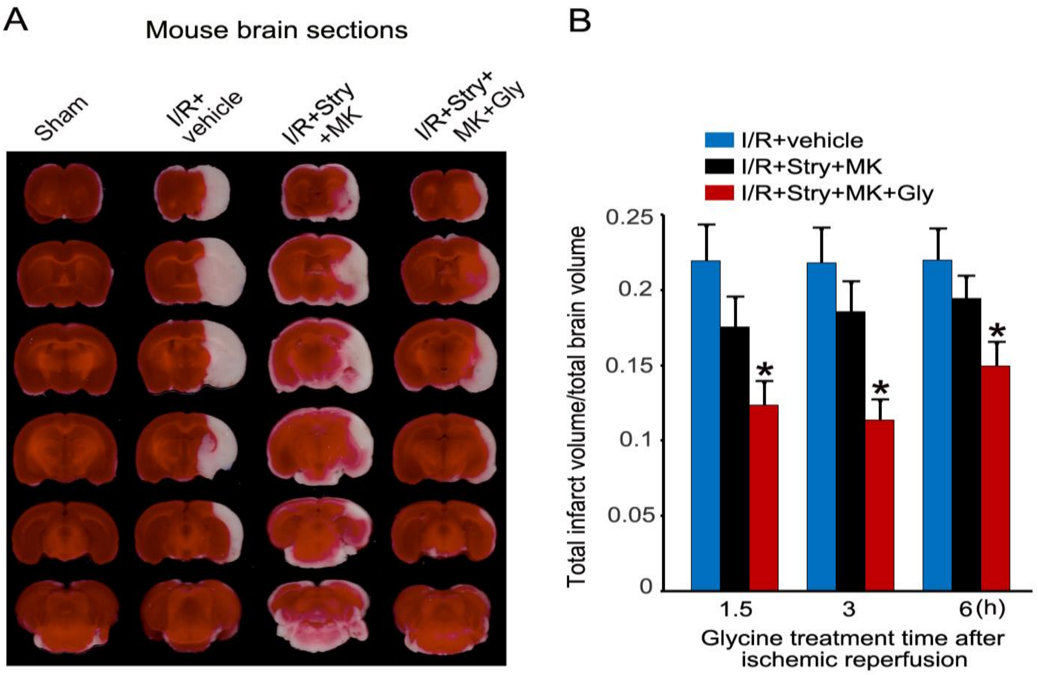

Figure S2. Glycine treatment reduces the infarct volume of ischemic mouse brain independent of glycine receptor activation and the channel activity of NMDARs. (**A**) Sample images of TTC strained-mouse brain sections collected at 24 h after ischemia onset in a mouse MCAo model. Glycine (100 μg/100 g, icv) was administered at 3 h following ischemia-reperfusion (I/R). At 30 min prior to glycine injection, MK-801 (8.0 μg/100 g, icv) and

strychnine (1.2 µg/100 g, icv) were injected. (**B**) Summarized quantification data of (A) indicate that glycine treatment at 1.5, 3, or 6 h following I/R reduces infarct volume after glycine receptors and NMDARs are inhibited (n = 6; ANOVA test, *P < 0.05 vs. I/R+Stry+MK). Stry: Strychnine; Gly: glycine; MK: MK-801
